# Review of the West Indian genus *Monotalla* Bechyné (Coleoptera, Chrysomelidae, Galerucinae, Alticini) with description of five new species

**DOI:** 10.3897/zookeys.505.9434

**Published:** 2015-05-25

**Authors:** Alexander S. Konstantinov, Adelita M. Linzmeier, Shawn M. Clark, Michael A. Ivie

**Affiliations:** 1 Systematic Entomology Laboratory, USDA, ARS, c/o Smithsonian Institution, P.O. Box 37012, National Museum of Natural History, Washington, DC, 20013-7012, USA; 2 Universidade Federal da Fronteira Sul–UFFS, Rua Edmundo Gaievski, 1000, 85.770-000, Realeza–PR, Brazil; 3 Monte L. Bean Life Science Museum, Brigham Young University, Provo, Utah 84602, USA; 4 Montana Entomology Collection, Marsh Lab Rm 50, 1911 West Lincoln Street, Montana State University Bozeman, MT 59717, USA

**Keywords:** New species, flea beetles, West Indies, leaf litter, moss

## Abstract

The West Indian genus *Monotalla* Bechyné is reviewed, redescribed and illustrated. Five new species are added: *Monotalla dominica* **sp. n.** (Dominica); *Monotalla lecticofolia* **sp. n.** (St. Lucia); *Monotalla maierae* **sp. n.** (St. Lucia); *Monotalla obrienorum* **sp. n.** (Grenada); and *Monotalla viridis* **sp. n.** (St. Lucia). A key to *Monotalla* species is provided.

## Introduction

*Monotalla* was originally proposed for two species, with *Monotalla guadeloupensis* Bechyné, 1956 as the genotype. It was later synonymized with *Pseudodibolia* Jacoby, 1891 by Scherer (1962). However, further studies revealed that it is substantially different from *Pseudodibolia* based on various features including the presence of only ten antennomeres while *Pseudodibolia* species have 11 (Savini and Furth 2001). The second species included in *Monotalla* by Bechyné (1956), *Monotalla nigrita* (Jacoby) originally proposed in the genus *Glyptina* LeConte, 1859, does not belong to *Monotalla*, although its generic placement is as yet unknown (Savini and Furth 2001).

The Guadeloupe record for *Monotalla guadeloupensis* is surely from the island of Basse-Terre, where the subsequent collections originate. None of the other islands that make up the Guadeloupe archipelago are high enough to harbor *Monotalla*. All specimens with good locality data are from elevations above 525m (on Basse-Terre) to nearly 800m (on St. Lucia).

*Monotalla* specimens have been collected by malaise trapping, berleseing leaf litter and moss, and beating a dead palm frond and unspecified vegetation (in the case of the O’Brien specimens), but in all cases, these activities were in unusual high elevation wet forests. Clearly, finding *Monotalla* requires special methods and efforts in unique and limited habitats. The Smithsonian Archibold-Breden Survey of Dominica, the longest and most richly funded entomological inventory of any West Indian Island (Peck 2006) did not yield a single specimen. The Piton Troumasse location on St. Lucia, which yielded 2 species and the largest series of specimens, is in cloud forest on a knife-edge ridge, a very difficult to reach habitat nearly completely covered in moss. Very few collectors exert the effort to reach these small, steep, wet, slippery and often cold localities. The St. Lucia locality was visited by teams from the West Indian Beetle Fauna Project 7 times over a 5 week period in 2009, during which time traps were deployed continuously and each time sifted litter was returned to the base for Berlese treatment. Another 18 localities in other representative habitat types on St. Lucia were given roughly the same level of effort, but produced zero *Monotalla*. That such effort was required to yield a handful of specimens may explain the absence of known species from other seemingly suitable but less studied islands lying between Basse-Terre and Grenada, namely St. Vincent and Martinique. Other of the Lesser Antillean Volcans north of Basse-Terre, from Saba to Montserrat, reach suitable elevations, but the tiny pockets in the very highest and wettest areas are still virtually unknown. Only Montserrat (Ivie et al. 2008) and Saba (Ivie and D. S. Sikes unpublished) have had any significant effort devoted to these islands, and as yet, no *Monotalla* specimens have been found.

To the west and north, extensive targeted moss sifting in the Greater Antilles by A.S. Konstantinov (Dominican Republic: 2004–2006, 2014 and Puerto Rico: 2008, 2014) did not reveal any *Monotalla* specimens, so the genus may indeed be limited to the Lesser Antilles.

## Material and methods

Dissecting techniques, measurements, and terminology follow Konstantinov (1998). Digital images were taken with an AxioZoom.V16 Zeiss microscope with a digital camera attached to it. Habitus illustration is produced with a technique described by Litwak and Harel (2013). Observation on the size of punctures on the vertex, pronotum and elytra was done under a Stemi SV11 Zeiss microscope with a Plan-Apochromat 1.6× objective with the light shining straight down on the surface. This makes punctures look generally larger than on the images taken with digital camera.

Specimens are deposited in the National Museum of Natural History, Smithsonian Institution, Washington DC, USA (USNM), Monte L. Bean Life Science Museum, Brigham Young University, Provo, Utah (BYUC), Natural History Museum, Basel, Switzerland (NHMB), and West Indian Beetle Fauna Project Collection, Montana State University, Bozeman, Montana (WIBF).

**Map 1.**
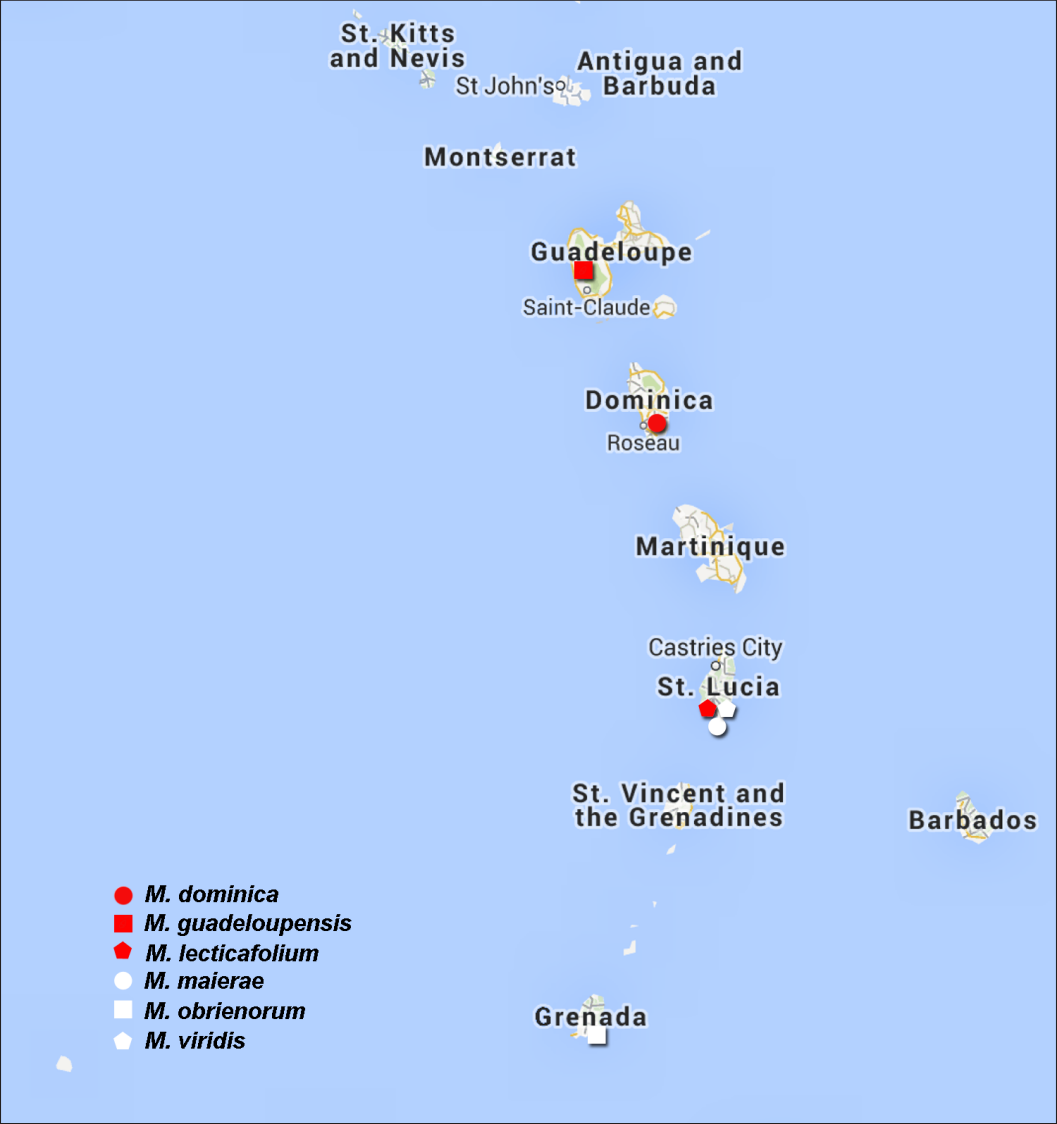
Distribution of *Monotalla* species in Lesser Antilles.

## Results

### Monotalla

Taxon classificationAnimaliaColeopteraChrysomelidae

Bechyné, 1956

Figs 1 , 2–6 , 7–12 , 13–17 , 18–21 , 22–26 , 27–31 , 32–35 , 36–41 , 42–44 , 45–49 , Map 1

Monotalla Bechyné, 1956: 588 (type species *Monotalla guadeloupensis* Bechyné, 1956: 588, original designation, type locality Guadeloupe).

#### Description.

Body length 1.24–1.45 mm, width 0.80–0.91 mm, oval, relatively convex in lateral view (1.70 times as long as thick). Color black, dark brown, greenish, bluish or lightly purple with metallic luster. Legs and antennae brown with femur and basal antennomeres darker than tibia and apical antennomeres. Venter light brown or amber in color.

Head moderately flat in lateral view. Frons and vertex forming slightly convex line in lateral view. Supraorbital pore absent. Antennal calli poorly developed, with all sulci around them absent. Supraorbital sulcus absent. Distance between eyes greater than transverse diameter of eye, much wider than transverse diameter of antennal socket. Frontal ridge wide, interiorly projecting beyond anterofrontal ridge. Anterofrontal ridge not separated from and as tall as frontal ridge. Eyes large, slightly protruding laterally, 0.57 times as wide as long. Vertex covered with evenly and widely spaced large and deep punctures. Labrum with four setiferous pores, apically slightly incised. Antenna with ten antennomeres. First antennomere wide, slightly wider and longer than second antennomere. Third antennomere nearly as long as, but much thinner than second. Antennomeres four and five much smaller than third. Remaining antennomeres much wider and longer than antennomere five.

Pronotum 2.19 times wider than long (measured in middle), without impressions, covered with large, deeply impressed punctures. Sides weakly rounded and narrowly explanate, with maximum width at base. Marginal anterolateral callosity situated obliquely to midline of beetle body. Posterolateral callosity not protruding laterally. Basal margin extending posteriorly, without distinct border in middle. Procoxal cavity widely open behind. Intercoxal prosternal process relatively narrow in middle, expanding posteriorly, extending beyond procoxae. Mesoscutellum flat, wider than long, apex sharply triangular, sides straight. Mesonotum short and wide with nearly horizontal prealar and postmedial projections. Mesocoxae mostly separated by both meso- and metasternum. Mesosternum not covered by metasternum, horizontal. Metasternum short, shorter than mesosternum.

Elytron widest near mid-length. Humeral callus absent. Elytral punctures arranged in rows. Punctures vary in size, smaller to much smaller than space between rows. Elytral apex narrowly rounded. Epipleura broad, slightly oblique, abruptly narrowing before apex. Mesothoracic wing present with poorly developed veins, except for radial cell and medial bar and spur.

Pro- and mesofemora more or less flat, widest near middle, canaliculated on ventral side facing tibiae. Metafemur robust, fairly flat dorsoventrally. Pro- and mesotibiae subcylindrical, slightly wider in distal 1/3, slanting towards tarsi, without spurs apically. Metatibia slightly curved in dorsal and lateral views, dorsal surface flat and deeply canaliculated near apex. Lateral (outer) margin dentate. Apical spur varying in length, but generally long. First metatarsomere attached before tibial apex, nearly as long as remaining metatarsomeres combined. Claw slightly appendiculate near base. Third tarsomere deeply incised.

Abdomen with five distinctly visible sternites. Apical sternite shorter than three preceding sternites combined, without appendages basally. Basal sternite without ridges in middle. Apical abdominal tergite without groove in middle, with a few long setae. Gut with long sclerotized folds.

Median lobe of aedeagus simple, slender, slightly curved in lateral view, more so at base and apex, without or with limited sculpture ventrally. Base with projection in middle.

Vaginal palpi with anterior sclerotizations merged together. Posterior sclerotizations of vaginal palpi oblique or straight at apex. Tignum gradually widening posteriorly before posterior membrane. Spermatheca without distinct border between receptacle and pump. Pump with long appendage at apex. Receptacle bent in basal half. Spermathecal duct long, straight basally, making one loop.

**Figure 1.**
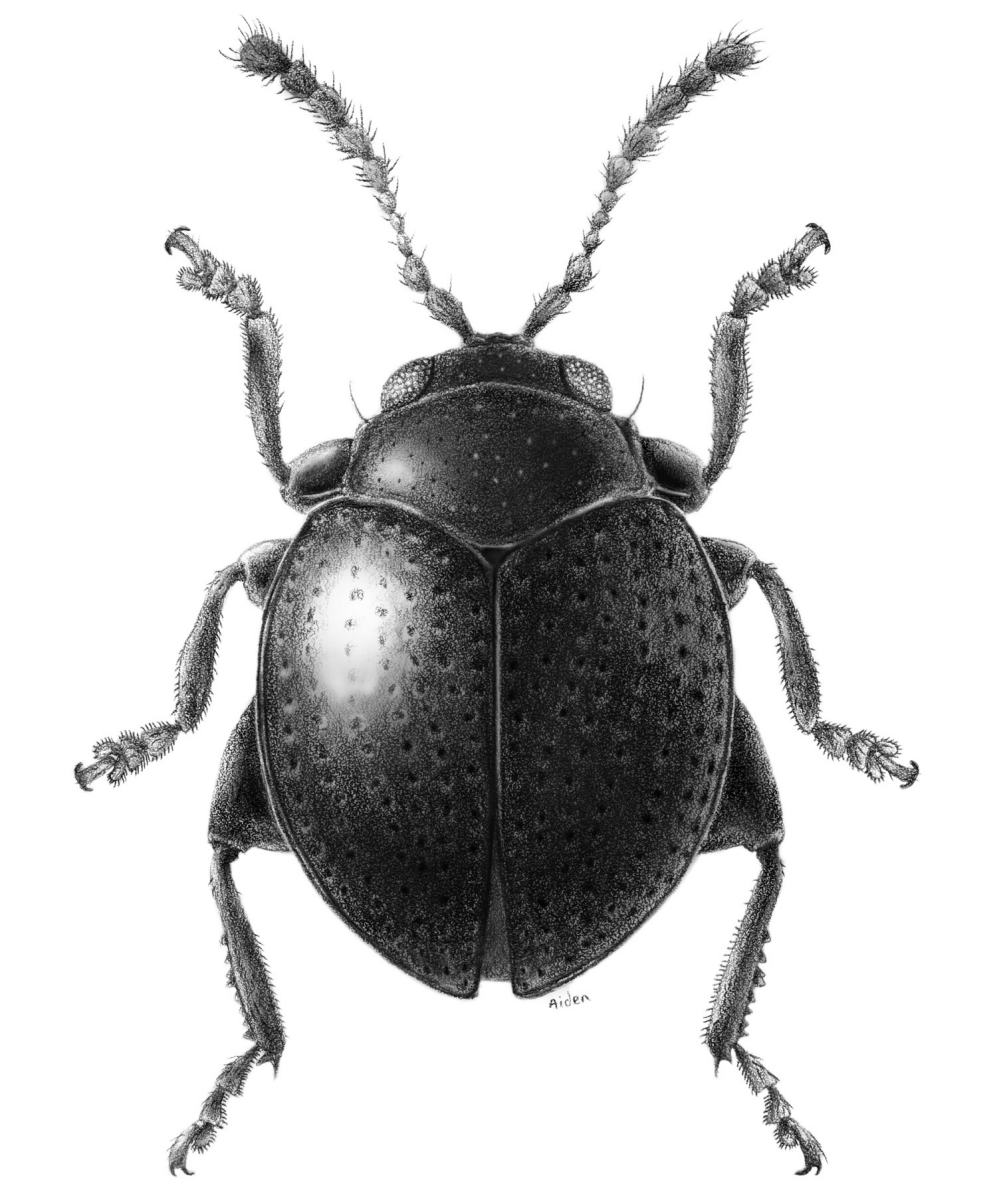
*Monotalla guadeloupensis*, dorsal habitus.

**Figures 2–6.**
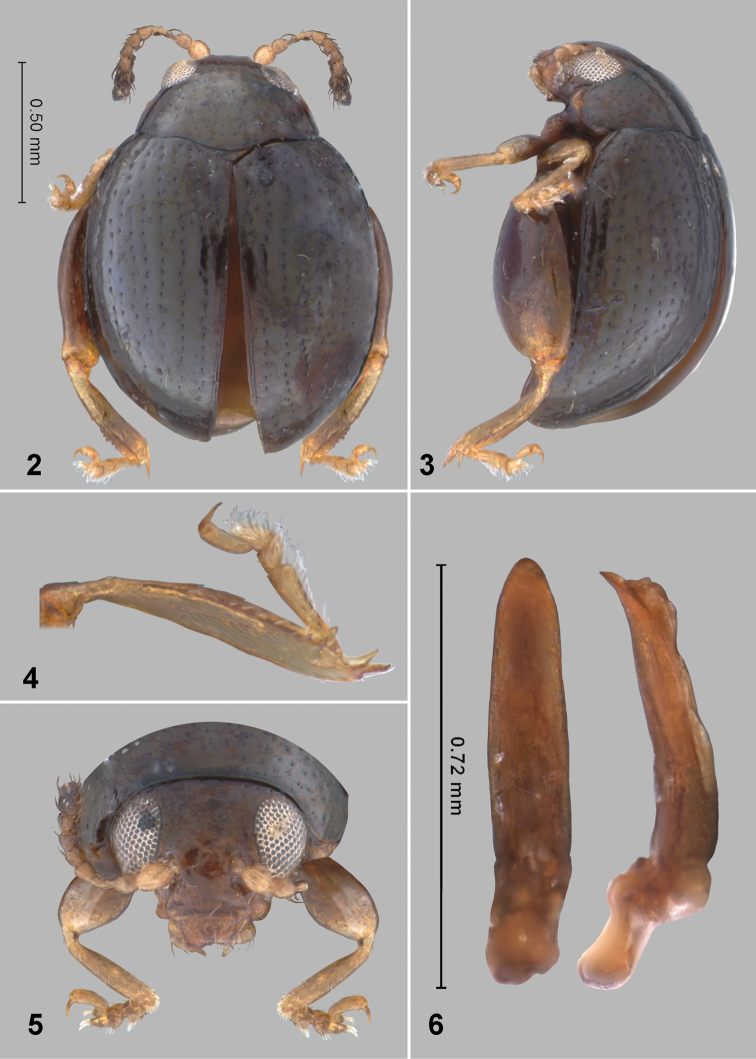
*Monotalla dominica*. **2** Dorsal habitus **3** Lateral habitus **4** Hind tibia and tarsi **5** Head, frontal view **6** Aedeagus, ventral and lateral views.

**Figures 7–12.**
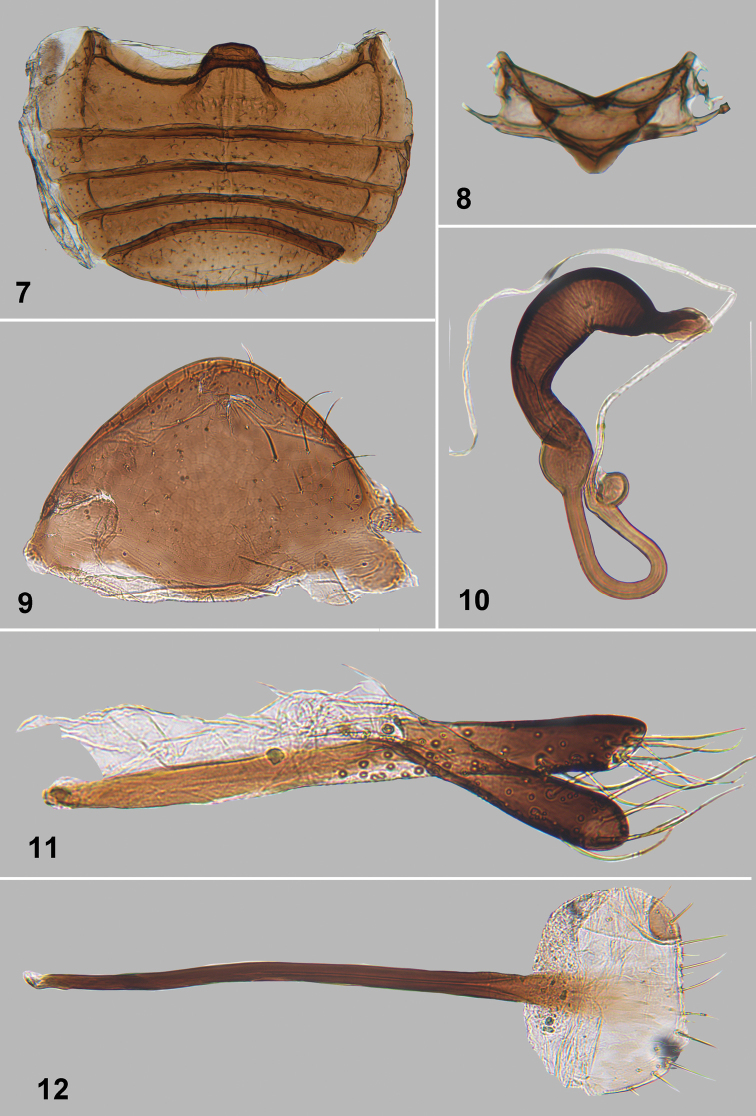
*Monotalla dominica*. **7** Abdominal ventrites **8** Mesotergite **9** Apical abdominal tergite **10** Spermatheca **11** Vaginal palpi **12** Tignum.

**Figures 13–17.**
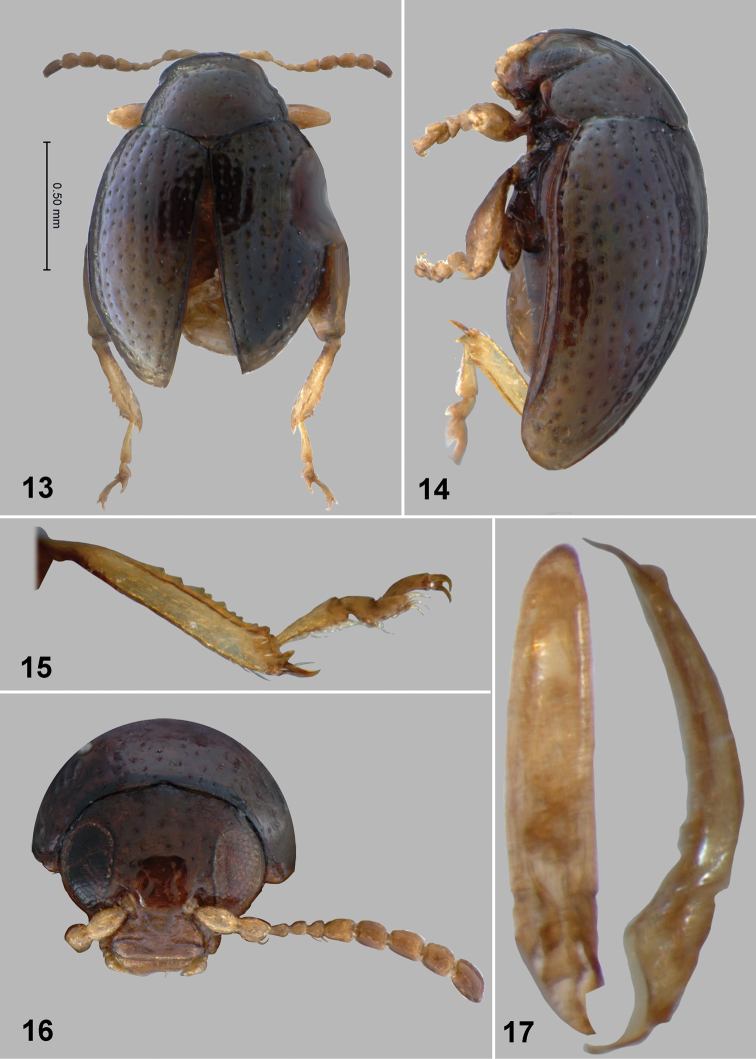
*Monotalla guadeloupensis*. **13** Dorsal habitus **14** Lateral habitus **15** Hind tibia and tarsi **16** Head, frontal view **17** Aedeagus, ventral and lateral views.

**Figures 18–21.**
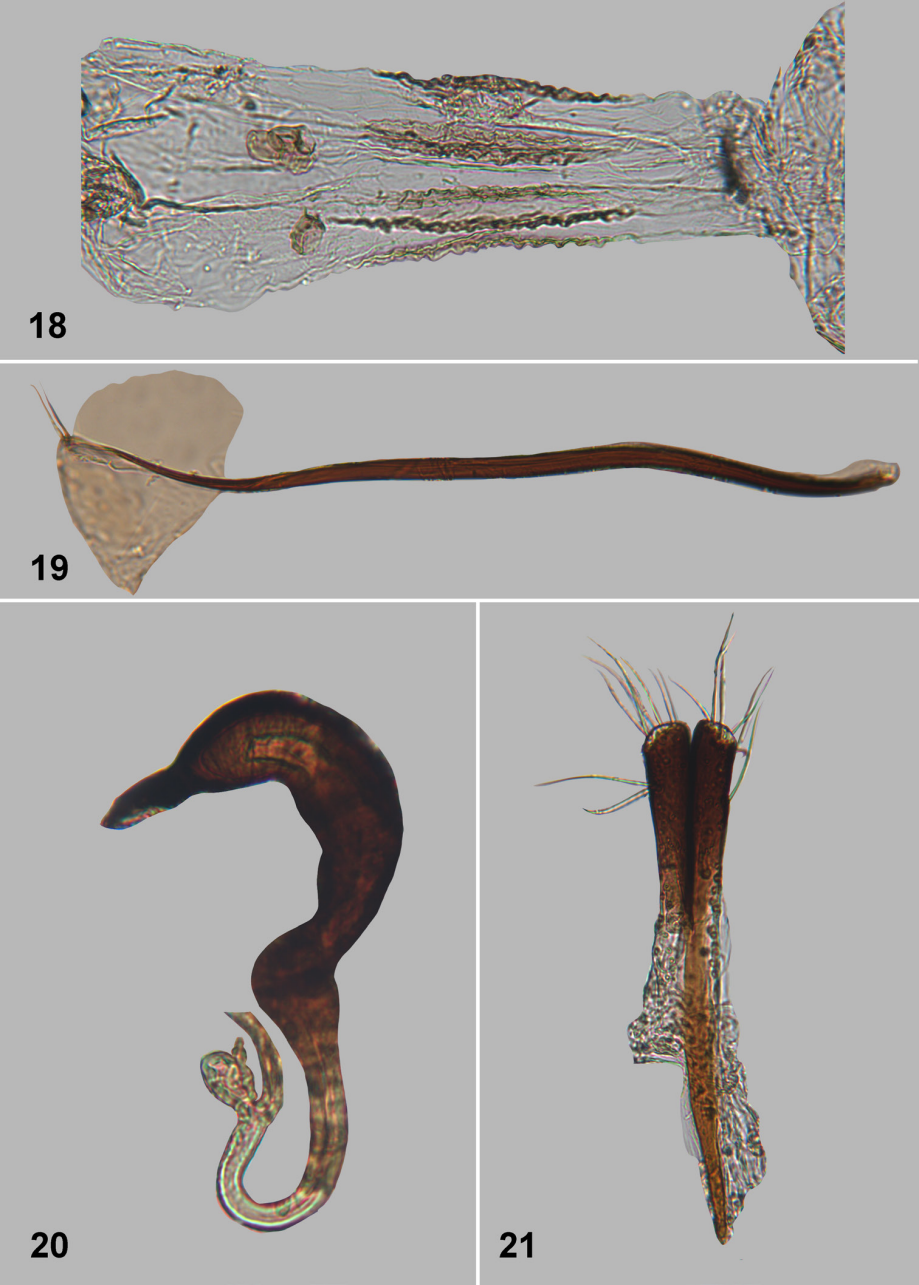
*Monotalla guadeloupensis*. **18** Gut, with scerotized folds **19** Tignum **20** Spermatheca **21** Vaginal palpi.

**Figures 22–26.**
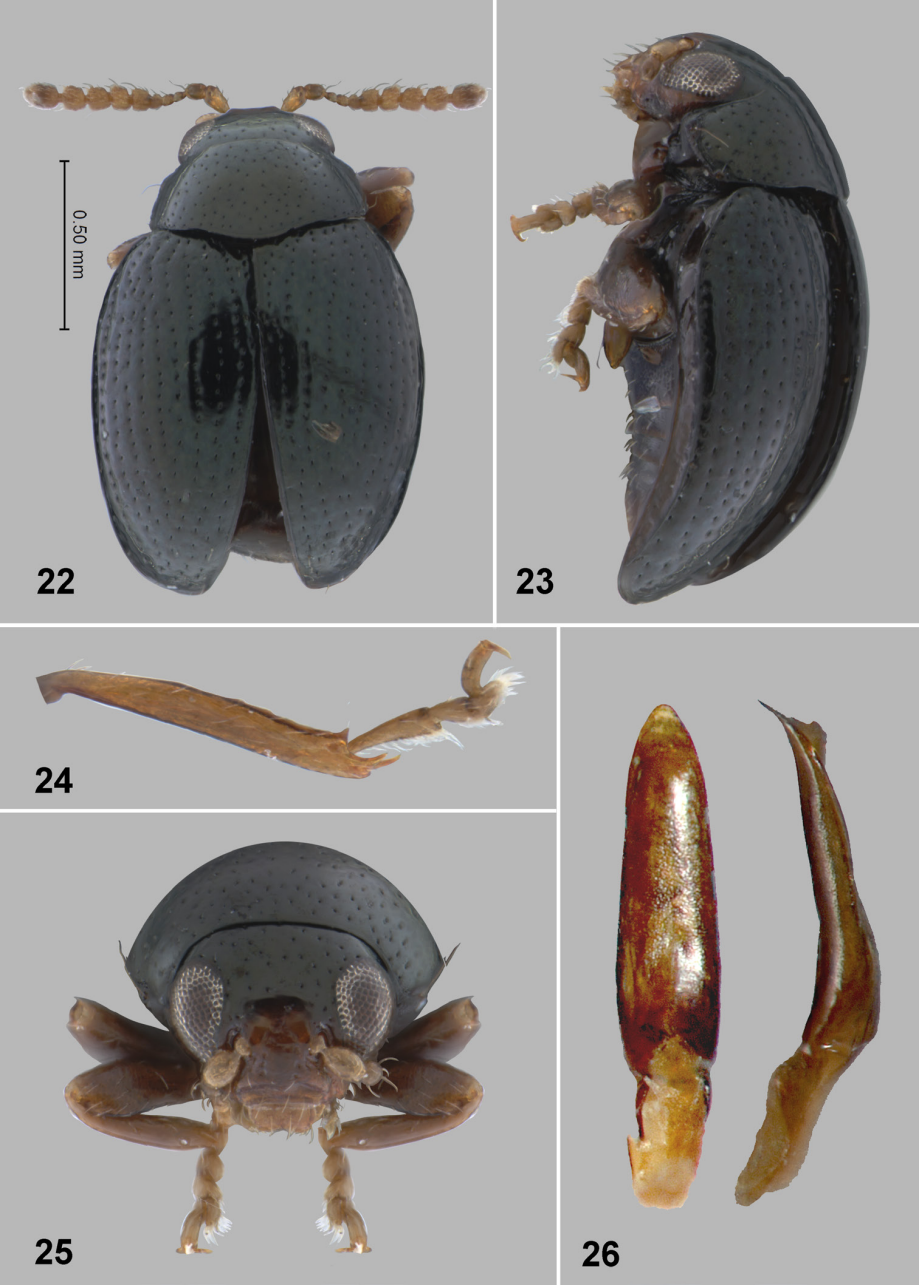
*Monotalla lecticafolium*. **22** Dorsal habitus **23** Lateral habitus **24** Hind tibia and tarsi **25** Head, frontal view **26** Aedeagus, ventral and lateral views.

**Figures 27–31.**
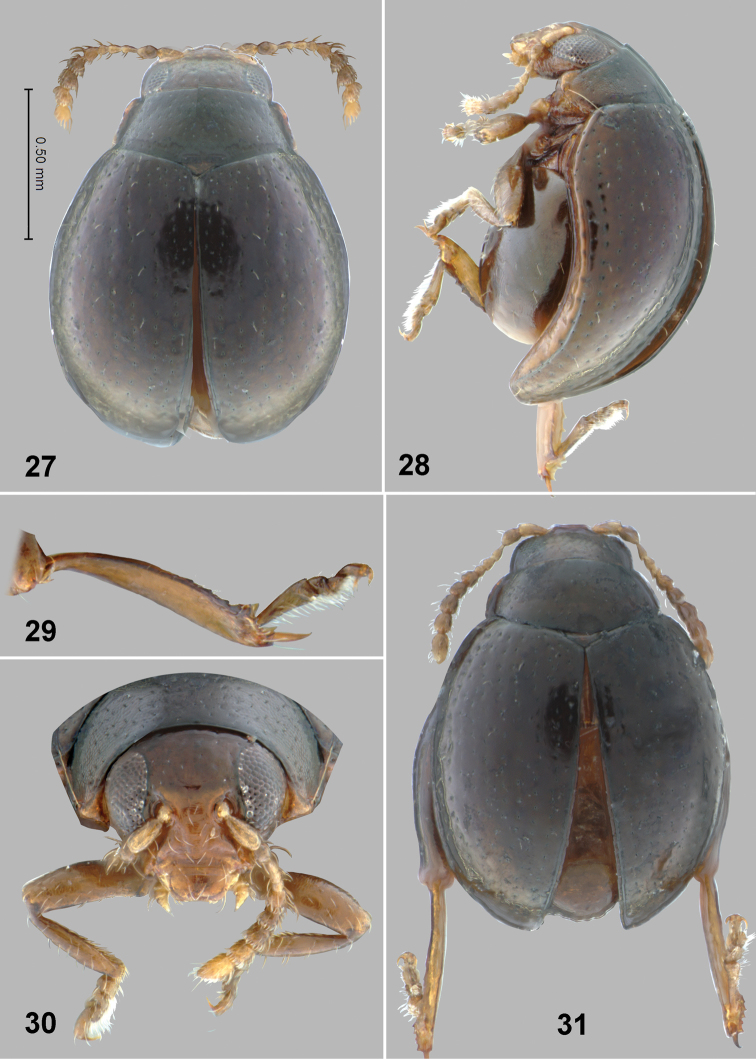
*Monotalla maierae*. **27** Holotype, dorsal habitus **28** Holotype, lateral habitus **29** Hind tibia and tarsi **30** Head, frontal view **31** Paratype, dorsal habitus.

**Figures 32–35.**
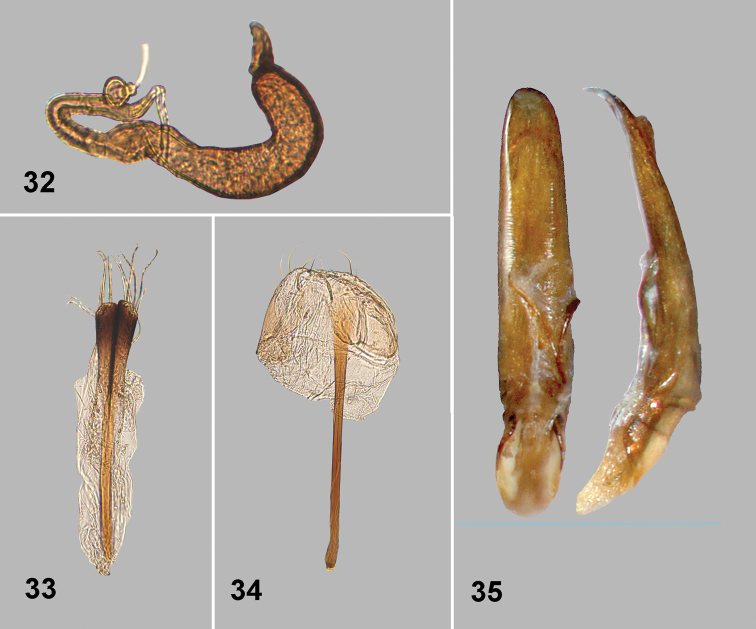
*Monotalla maierae*. **32** Spermatheca **33** Vaginal palpi **34** Tignum **35** Aedeagus, ventral and lateral views.

**Figures 36–41.**
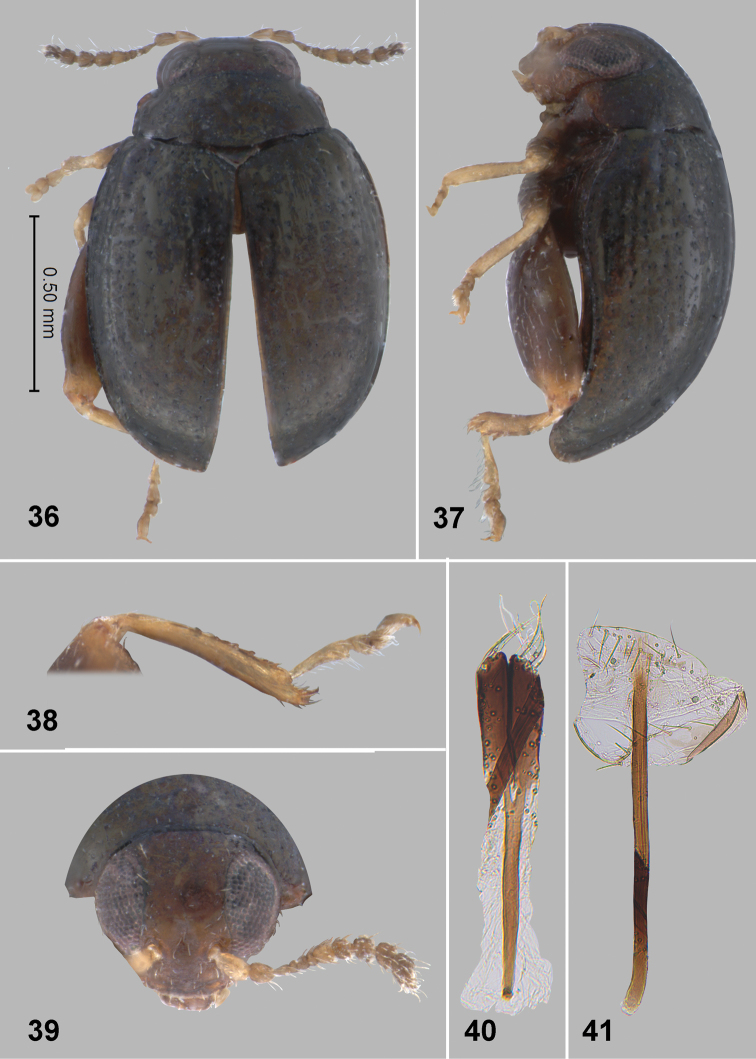
*Monotalla obrienorum*. **36** Dorsal habitus **37** Lateral habitus **38** Hind tibia and tarsi **39** Head, frontal view **40** Vaginal palpi **41** Tignum.

**Figures 42–44.**
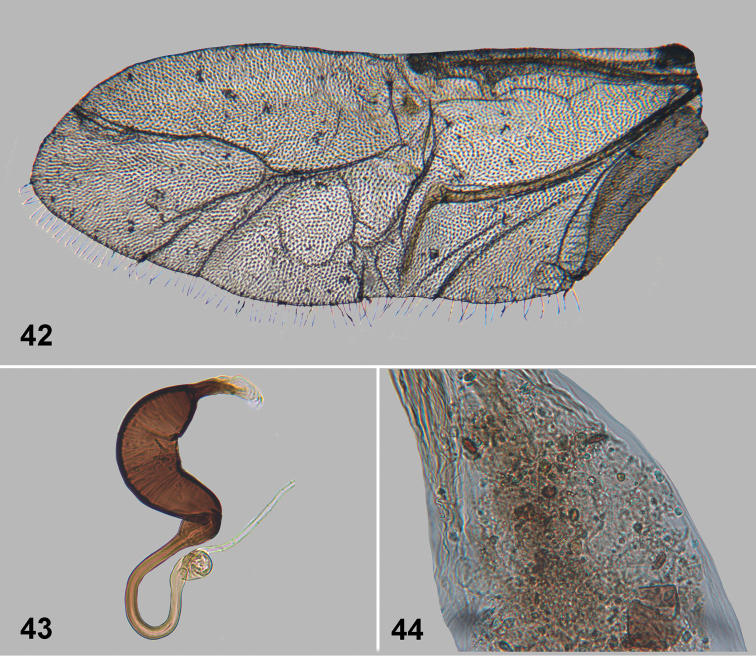
*Monotalla obrienorum*. **42** Hind wing **43** Spermatheca **44** Gut.

**Figures 45–49.**
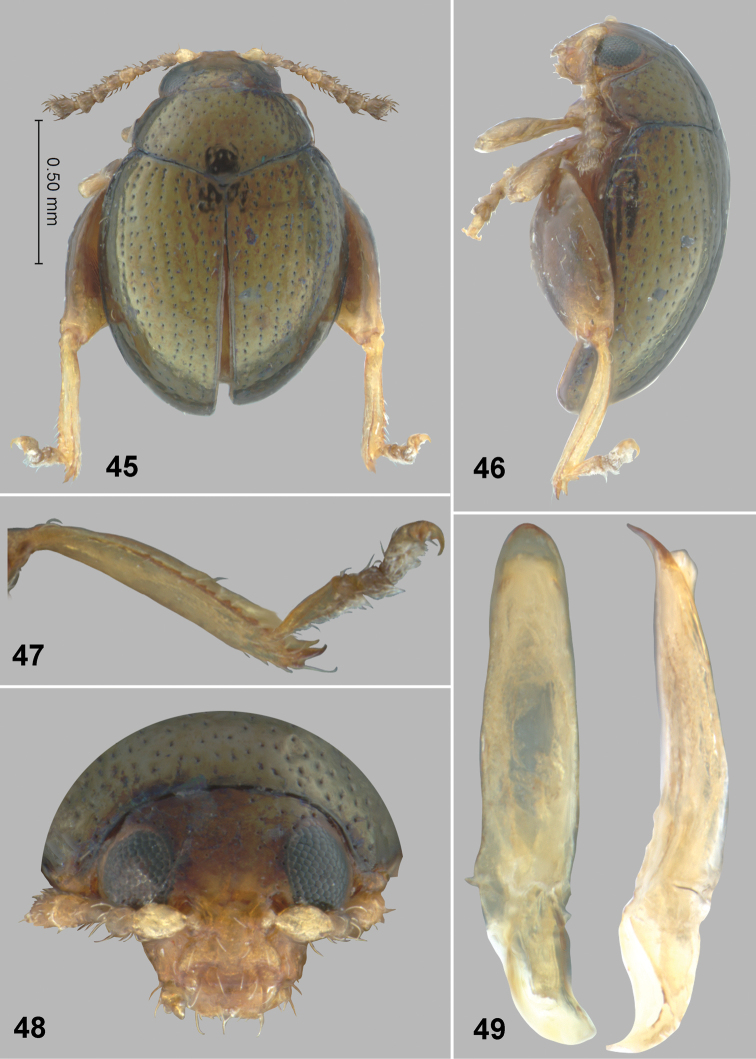
*Monotalla viridis*. **45** Dorsal habitus **46** Lateral habitus **47** Hind tibia and tarsi **48** Head, frontal view **49** Aedeagus, ventral and lateral views.

### Monotalla dominica

Taxon classificationAnimaliaColeopteraChrysomelidae

Konstantinov, Linzmeier, Clark & Ivie sp. n.

http://zoobank.org/567042A3-5290-412F-8495-196EFE856DE1

Figs 2–6 , 7–12

#### Description.

Body length: 1.28–1.38 mm, width: 0.84–1.10 mm. Color blackish with light bluish luster. Ventral side and appendages light brown or amber. Distance between eyes about 2 times wider than transverse diameter of eye. Diameter of punctures on vertex smaller than distance between them. Pronotal punctures sparse, slightly elongate in shape, their transverse diameter much smaller than distance between them. Elytral punctures relatively large, forming well developed rows. Distance between punctures in rows about as great as their diameter. Distance between rows much greater than diameter of punctures. Lateral (outer) side of metatibia deeply and coarsely dentate. Metatibial spur curved, nearly as long as denticle situated on apex of inner side of metatibia. Posterior sclerotizations of vaginal palpi nearly parallel-sided, straight at apex. Posterior sclerotization of tignum gradually widening posteriorly, abdominal sternite 8 with two sclerotized spots near apex. Spermatheca with relatively long middle part of receptacle, with internal side forming nearly 90 degree angle.

#### Comments.

*Monotalla dominica* can be separated from other *Monotalla* by its short metatibial spur situated on the apex of the inner side of the metatibia that barely projects beyond apicomedial denticle.

#### Etymology.

This species is named after the island where it occurs.

#### Type material.

Holotype, female: Dominica, ca 2600’, Morne Trois Pitons N.P., Freshwater Lake, 17-8-1986, C.W. & L. O’Brien (WIBF, to be deposited in the USNM). Paratype, female, the same label as the holotype (WIBF). Paratype female: Dominica: St. Paul Parish; Morne Trois Piton N.P.; Trail to Middleham Falls; 27May-05JUNE 2011; Santee Malaise (WIBF). Data not on the labels indicate this locality is at 724m (M.Ivie, unpublished) (WIBF). The specimens are provided with one additional label (Holotype or Paratype respectively) *Monotalla dominica* Konstantinov et al. 2015.

### Monotalla guadeloupensis

Taxon classificationAnimaliaColeopteraChrysomelidae

Bechyné, 1956

Figs 1 , 13–17 , 18–21

Monotalla guadeloupensis Bechyné, 1956: 588 (type locality Guadeloupe, type NHMB), Savini and Furth 2001: 907 (status restored).

#### Description.

Body length: 1.32–1.35 mm, width: 1.05–1.10 mm. Color blackish with light bluish metallic luster. Ventral side and appendages light brown or amber. Distance between eyes about 2 times wider than transverse diameter of eye. Punctures on vertex about as large as distance between them. Pronotal punctures sparce, slightly elongate in shape, their transverse diameter much smaller than distance between them. Elytral punctures relatively large, forming well developed rows. Distance between punctures in rows about as great as their diameter. Distance between rows at base of elytra about as great as diameter of punctures. Distance between rows in middle of elytra greater than diameter of punctures. Lateral (outer) side of metatibia deeply and coarsely dentate. Metatibial spur curved, projects beyond denticle situated on apex of inner side of metatibia. Aedeagus slender, with shallow and wide impression ventrally. Posterior sclerotizations of vaginal palpi slightly widening apically, straight at apex. Posterior sclerotization of tignum gradually narrowing posteriorly, abdominal sternite 8 without sclerotized spots near apex. Spermatheca with relatively long middle part of receptacle, with internal side forming few folds.

#### Comments.

*Monotalla guadeloupensis* is the type species of *Monotalla*. In the key to *Monotalla* species it ends up in the same couplet with *Monotalla lecticafolium*. It can be separated by the following characters: punctures on vertex about as large as distance between them; aedeagus slender, with shallow and wide impression ventrally. In *Monotalla lecticafolium*, punctures on vertex are much smaller than distance between them and aedeagus is robust, without impression ventrally.

#### Type material.

Holotype, male: 1) Guadeloupe; 2) 1953 Coll Heikertinger; 3) Holotype *Monotalla guadeloupensis* J. Bechyné det. 1956. (NHMB).

#### Material examined.

Guadeloupe: Basse Terre, Mam[elles] de Pigeon, 600-700m, 16°10.668N, 61°44.152W, 21 Aug 2005, M.A. Ivie, beating dead palm frond (male WIBF, female USNM). Guadeloupe: Basse T. Gourbeyre, Palmiste, 05-20 Jan 2003, J. Touroult colr. (male WIBF).

### Monotalla lecticofolia

Taxon classificationAnimaliaColeopteraChrysomelidae

Konstantinov, Linzmeier, Clark & Ivie sp. n.

http://zoobank.org/6F087FA1-1B04-4FC5-8EA8-C5B6E59C550D

Figs 22–26

#### Description.

Body length: 1.42–1.43 mm, width: 1.98–1.10 mm. Color blackish with light bluish metallic luster. Ventral side and appendages light brown or amber. Distance between eyes about 2 times wider than transverse diameter of eye. Punctures on vertex much smaller than distance between them. Pronotal punctures sparse, slightly elongate in shape, their transverse diameter much smaller than distance between them. Elytral punctures relatively large, forming well developed rows. Distance between punctures in rows about as great as their diameter. Distance between rows greater than diameter of punctures. Lateral side of metatibia deeply and coarsely dentate. Apicomedial metatibial denticle shorter than metatibial spur. Aedeagus robust, without impression ventrally.

#### Comments.

In the key to *Monotalla* species *Monotalla lecticofolia* ends up in the same couplet with *Monotalla guadeloupensis*. It can be separated by the following characters: punctures on vertex much smaller than distance between them and aedeagus robust, without impression ventrally. In *Monotalla guadeloupensis*, punctures on vertex about as large as distance between them; aedeagus slender, with shallow and wide impression ventrally.

#### Etymology.

This species name comes from Latin words “*lectico*” to collect something from somewhere and “*folia*” leaf.

Type material: Holotype, male: 1) St. Lucia: Piton Troumasse trap site. 793m, 13.8535°N, 61.0098°W, 22–30 JUNE 2009 malaise, C. A. Maier & M. L. Gimmel (WIBF, to be deposited in the USNM). Paratypes, male, the same label as the holotype (WIBF). The specimens are provided with one additional label (Holotype or Paratype respectively) *Monotalla lecticofolia* Konstantinov et al. 2015.

### Monotalla maierae

Taxon classificationAnimaliaColeopteraChrysomelidae

Konstantinov, Linzmeier, Clark & Ivie sp. n.

http://zoobank.org/641A4308-BB17-4EC7-9537-1EEE8566B7D2

Figs 27–31 , 32–35

#### Description.

Body length: 1.32–1.35 mm, width: 1.08–0.90 mm. Elytra with purplish luster. Ventral side and appendages light brown or amber. Distance between eyes about 2 times wider than transverse diameter of eye. Punctures on vertex poorly defined, mostly smaller than distance between them. Pronotal punctures sparse, slightly elongate in shape, their transverse diameter much smaller than distance between them. Elytral punctures relatively small, forming rows. Distance between punctures in rows lesser than or equal to their diameter. Distance between rows greater than diameter of punctures. Lateral side of metatibia with short evenly spaced denticles. Metatibial spur straight, strongly projecting beyond denticle situated on apex of inner side of metatibia. Aedeagus slender, with shallow and wide impression ventrally. Posterior sclerotizations of vaginal palpi slightly widening apically, straight at apex. Posterior sclerotization of tignum gradually narrowing posteriorly, abdominal sternite 8 without sclerotized spots near apex. Spermatheca with relatively long middle part of receptacle, with internal side slightly bend.

#### Comments.

*Monotalla maierae* can be separated from all other of *Monotalla* species by the purplish elytra. In addition, *Monotalla maierae* differs from most *Monotalla* based on small and sparse pronotal and elytral punctures.

#### Etymology.

We name this species after C. A. Maier who collected three of five new species described in this paper.

#### Type material.

Holotype, male: 1) St. Lucia: Piton Troumasse trap site. 793m, 13.8535°N, 61.0098°W, 17 JUNE 2009. moss berlese C. A. Maier (WIBF, to be deposited in the USNM). Paratypes 2 males, the same label as holotype (1- WIBF, 1- BYUC). Paratypes 2 females: 1) St. Lucia: Piton Troumasse trap site. 793m, 13.8535°N, 61.0098°W, 22 JUNE 2009. litter berlese C. A. Maier (1 - USNM, 1 - WIBF). The specimens are provided with one additional label (Holotype or Paratype respectively) *Monotalla maierae* Konstantinov et al. 2015.

### Monotalla obrienorum

Taxon classificationAnimaliaColeopteraChrysomelidae

Konstantinov, Linzmeier, Clark & Ivie sp. n.

http://zoobank.org/95E5FA0F-2563-4F27-A306-7B8E652E11D2

Figs 36–41 , 42–44

#### Description.

Body length: 1.18–1.25 mm, width: 0.91–0.77 mm. Elytra with blackish and bluish luster. Ventral side and appendages light brown or amber. Distance between eyes about 1.4–1.5 times wider than transverse diameter of eye. Punctures on vertex well defined, sparse, smaller than distance between them. Pronotal punctures sparse, slightly elongate in shape, their transverse diameter much smaller than distance between them. Elytral punctures larger than those on pronotum, forming rows. Distance between punctures in rows smaller than or as great as their diameter. Distance between rows greater than diameter of punctures. Lateral side of metatibia with short evenly spaced denticles. Metatibial spur curved, projecting beyond denticle situated on apex of inner side of metatibia. Posterior sclerotizations of vaginal palpi with slightly sinusoidal lateral side, straight at apex. Tignum more or less parallel sided, abdominal sternite 8 without sclerotized spots near apex. Spermatheca with relatively long middle part of receptacle, with internal side slightly bend.

#### Comments.

*Monotalla obrienorum* can be separated from all other *Monotalla* species based on the distance between eyes being 1.40–1.50 times greater than the transverse diameter of the eye.

#### Etymology.

We name this species after Charles W. & Lois B. O’Brien who collected two of five new species described in this paper.

#### Type material.

Holotype, female: 1) Grenada, Grand Etang, N.P. Mt. Qua Qua Tr., 10-IX-1991 C.W. & L. B. O’Brien (BYUC). Paratypes, 3 females, the same label as the holotype (1- BYUC, 1- USNM, 1 - WIBF). Paratype, female: 1) Grenada, St. John P, 1 mi E. Gouyave, 5.IX.1991. C.W. & L. B. O’Brien (BYUC). The specimens are provided with one additional label (Holotype or Paratype respectively) *Monotalla obrienorum* Konstantinov et al. 2015.

### Monotalla viridis

Taxon classificationAnimaliaColeopteraChrysomelidae

Konstantinov, Linzmeier, Clark & Ivie sp. n.

http://zoobank.org/1DEB2FE1-9279-4166-A47B-97D450843E31

Figs 45–49

#### Description.

Body length: 1.25 mm, width: 0.88 mm. Elytron with light greenish luster. Ventral side and appendages light brown or amber. Distance between eyes slightly less than 2 times wider than transverse diameter of eye. Punctures on vertex well defined, sparse, smaller than distance between them. Pronotal punctures sparse, slightly elongate in shape, their transverse diameter much smaller than distance between them. Elytral punctures slightly larger than those on pronotum, forming rows. Distance between punctures in rows smaller than or as great as their diameter. Distance between rows greater than diameter of punctures. Lateral side of metatibia with relatively large denticles. Metatibial spur curved, projecting beyond denticle situated on apex of inner side of metatibia. Aedeagus more or less robust, without impression ventrally, nearly straight in lateral view.

#### Comments.

*Monotalla viridis* can be separated from all other *Monotalla* species based on the light greenish color of the elytra.

#### Etymology.

This species is named after its light greenish color.

#### Type material.

Holotype, male: 1) St. Lucia: Piton St. Esprit trap site. 571m, 13.8493°N, 60.9795°W, 29 MAY 2009. ex. tree moss C. A. Maier; 2) Holotype *Monotalla viridis* Konstantinov et al. 2015 (WIBF, to be deposited in the USNM).

### Key to *Monotalla* species

**Table:** 

1	Elytron with light greenish luster. St. Lucia	***Monotalla viridis* sp. n.**
–	Elytron with bluish, blackish or purplish luster	**2**
2(1)	Metatibial spur barely projecting beyond denticle situated on apex of inner side of metatibia. Dominica	***Monotalla dominica* sp. n.**
–	Metatibial spur strongly projects beyond denticle situated on apex of inner side of metatibia	**3**
3(2)	Elytron with purplish luster. St. Lucia	***Monotalla maierae* sp. n.**
–	Elytron with bluish or blackish luster	**4**
4(3)	Distance between eyes 1.40–1.50 times greater than transverse diameter of eye. Grenada	***Monotalla obrienorum* sp. n.**
–	Distance between eyes 2.01–2.15 times greater than transverse diameter of eye	**5**
5(4)	Punctures on vertex about as large as distance between them. Aedeagus slender, with shallow and wide impression ventrally. Basse-Terre	***Monotalla guadeloupensis* Bechyné, 1956**
–	Punctures on vertex much smaller than distance between them. Aedeagus robust, without impression ventrally. St. Lucia	***Monotalla lecticofolia* sp. n.**

## Supplementary Material

XML Treatment for Monotalla

XML Treatment for Monotalla dominica

XML Treatment for Monotalla guadeloupensis

XML Treatment for Monotalla lecticofolia

XML Treatment for Monotalla maierae

XML Treatment for Monotalla obrienorum

XML Treatment for Monotalla viridis
